# Evaluation of a Team-Based Learning Tutor Training Workshop on Research and Publication Ethics by Faculty and Staff Participants

**DOI:** 10.3352/jeehp.2009.6.5

**Published:** 2009-12-20

**Authors:** Young-Su Ju

**Affiliations:** Department of Social Medicine, College of Medicine, Hallym Universitye and Hallym University Sacred Heart Hospital, Anyang, Korea.

**Keywords:** Team-Based Learning, Research Ethics, Publication Ethics, Faculty Training, Course Evaluation

## Abstract

A team-based Learning (TBL) tutor training workshop on research and publication ethics was offered to 8 faculty members and 3 staff at Hallym University in 2009. To investigate the effect of the workshop and any attitude changes, a questionnaire survey was performed after the 8-hr course. Questions in four categories-general course content, change in attitudes toward research and publication ethics, the TBL format, and an open-ended question about the course--were included. Participants responded positively to all items on general course content. There was a positive change in attitude on research and publication ethics. Participants also responded positively to six items on team-based learning. The overall positive response to the workshop on research and publication ethics suggested the effectiveness of this kind of TBL tutor training course for university faculty and staff.

Team-based learning (TBL) on research and publication ethics has been required of incoming faculty at the College of Medicine, Hallym University since February 2007 with positive results. For the continuation and expansion of this kind of faculty training, tutor training for TBL on research and publication ethics was suggested for university faculty beyond medical professors. Trained faculty members will be able to participate as tutors in graduate student education as well as resident training in the hospital. This paper introduces the curriculum of the workshop for TBL tutors on research and publication ethics and describes the evaluation of the course by participants.

In June 2009, the tutor training course for TBL on research and publication ethics was opened as a one-day 8 hr course. The timetable was as follows:

1. Research ethics on animal experiments
  Submission and review process of the animal experiment proposal
2. Research and publication ethics TBL
  Fabrication, falsification, and plagiarism
  Duplicate publication and authorship misconduct
3. Clinical research ethics
  Ethical consideration and the Institutional Review Board (IRB)
  Case discussion
  Exercise on reviewing a research proposal
4. How to organize and maintain an IRB in the institute

Four faculty members participated as tutors in the course. Participants consisted of 8 faculty members from bioscience, audiology, electrical engineering, nursing, radiology, psychiatry, family medicine, and hemato-oncology and 3 staff from the research support department and the laboratory animal center. The curriculum consisted of a brief lecture, an exercise, an introduction to the TBL training method, and a case discussion. TBL consists of an individual readiness assurance test, group readiness assurance test, and further application. First, individual participants try to answer items. After that, group members discuss the items together and select only one correct answer to each item. The tutor leads the participants to discuss the correct answers and their background reasoning. Further application is suggested through open-ended questions. This is intended to lead to advanced thinking and deep reasoning. Only 2 of the 8 hours were devoted to TBL. The rest of the time was focused on knowledge and skill acquisition on research and publication ethics.

To obtain feedback from participants, a 5-point Likert scale questionnaire with 5 being "strongly agree" and 1 being "strongly disagree" was distributed at the end of the workshop. The questionnaire used was derived from that of Kim [1]. It consisted of four categories: general course content (7 items); attitude toward research and publication ethics (6 items); the TBL format (5 items); and an open-ended question about the course (1 item). The items and summary of results are shown in Tables 1, 2, and 3. Mean and median responses to each item and the percentage of strongly positive responses (≥4) to each item are described.

To every items of questionnaire, there was a positive response. Out of 7 items on general course content, "the importance of learning materials" was the most positive item. There was a positive change in attitude on research and publication ethics. Out of 6 items on attitude toward research and publication ethics, "knowledge on the research and publication ethics increased"generated the most positive response. TBL format also showed a positive response, except for the item "increase in workload to participate in the class." Out of 5 items on the TBL format, "communication with the tutor" was the most positive response. Open-ended answers are as follows: It was good opportunity to understand research and publication ethics; It was important content; This is a useful program to apply to education; I will apply this program in my institute; It broadens my view of research and publication ethics.

The results of this evaluation showed that this kind of workshop on TBL tutor training was meaningful for the faculty and staff to understand the content of research and publication ethics and TBL procedures. A change in attitude on research and publication ethics appeared to occur in this workshop, and TBL tutor training might be another good basis for supporting the value of this workshop. In the future, they will be able to work as volunteer TBL tutors of for graduate students, residents, or peer faculty members not only in a special program but also as part of the classes they already teach. s. All faculty members, graduate students, and residents should be required to participate in a research and publication ethics training program since duplicate publication and other misconduct should be prevented [2]. TBL may be one good method to encourage active participation and true learning in such a program

## Figures and Tables

**Table 1:**
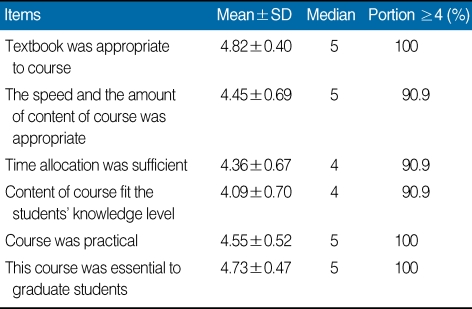
Participants' responses to six items addressing general satisfaction with the tutor training workshop for team-based learning on research and publication ethics, Hallym University, 2009

**Table 2:**
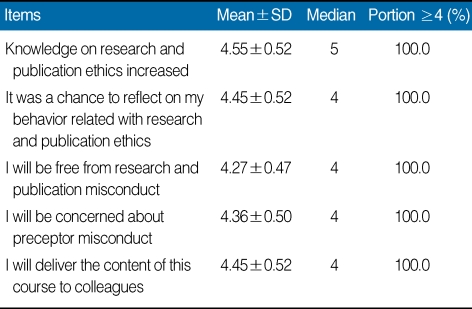
Results of participant responses to five items on their changes in attitude toward research and publication ethics during the tutor training workshop on research and publication ethics, Hallym University, 2009

**Table 3:**
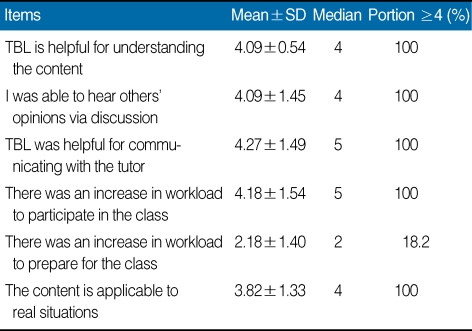
Results of participant responses to six items on teambased learning on research and publication ethics in the tutor training workshop on research and publication ethics, Hallym University, 2009
